# Comparison of Remimazolam–Flumazenil versus Propofol for Recovery from General Anesthesia: A Systematic Review and Meta-Analysis

**DOI:** 10.3390/jcm12237316

**Published:** 2023-11-26

**Authors:** Quantong Wu, Fuchao Xu, Jie Wang, Ming Jiang

**Affiliations:** Nanjing Drum Tower Hospital Clinical College of Nanjing Medical University, Nanjing 211166, China; quantongwu@stu.njmu.edu.cn (Q.W.); 2021121985@stu.njmu.edu.cn (F.X.); phobia1834@stu.njmu.edu.cn (J.W.)

**Keywords:** remimazolam, flumazenil, propofol, sedation, anesthesia, recovery, ERAS, systematic review, meta-analysis

## Abstract

(1) Purpose: to systematically evaluate the recovery following sedation and anesthesia with remimazolam combined with flumazenil in comparison to propofol. (2) Methods: Electronic databases, including PubMed, Embase, Web of Science, and the Cochrane Library, were systematically searched from their inception up to 22 October 2023. Included in this analysis were randomized controlled trials (RCT) that compared remimazolam–flumazenil with propofol for the recovery from sedation and anesthesia in adults. The risk of bias was assessed using the Cochrane risk of bias tool. Pooled risk ratios (RR) or mean differences (MD) along with their corresponding 95% confidence intervals (CI) were calculated using either fixed-effects or random-effects models, and the results were visualized in forest plots. (3) Results: Nine RCTs involving 745 patients who underwent general anesthesia in three different countries were included. Compared to propofol, the remimazolam–flumazenil combination shortened the emergence time (MD = −4.34 min, 95% CI = [−6.88, −1.81], *p* = 0.0008, low certainty), extubation time (MD = −4.26 min, 95% CI = [−6.81, −1.7], *p* = 0.0011, low certainty), and the post-anesthesia care unit (PACU) stay (MD = −4.42 min, 95% CI = [−7.45, −1.38], *p* = 0.0044, low certainty), while reducing the incidence of respiratory depression (RR = 0.2, 95% CI = [0.04, 0.89], *p* = 0.03, high certainty) after general anesthesia. However, this combination was associated with a higher incidence of re-sedation (RR = 4.15, 95% CI = [1.31, 13.13], *p* = 0.01, moderate certainty). (4) Conclusions: Based on the existing evidence, the combination of remimazolam and flumazenil accelerates recovery from general anesthesia and lowers the risk of respiratory depression compared to propofol. However, it is important to consider the higher risk of re-sedation when using this combination in clinical practice. Due to limitations in the quality of the evidence, it is advisable to interpret the results of meta-analyses with caution.

## 1. Introduction

Propofol is indisputably the most frequently utilized intravenous anesthetic, having been administered in over one billion surgical procedures on human patients since its inception in clinical practice [1]. Apart from its well-documented advantages such as rapid onset, smooth induction, and quick recovery, it also exhibits less-familiar yet valuable properties, including antiemetic, anti-inflammatory, antioxidant, organ-protective, and analgesic effects [2]. Since its introduction into clinical practice three decades ago, propofol has gained widespread popularity across the globe, catalyzing a global surge in the adoption of total intravenous anesthesia. The developer of propofol, John (Iain) Baird Glen, BVMS, PhD, was honored with the 2018 Lasker Clinical Research Award [3]. Nonetheless, propofol is not devoid of shortcomings, including injection-related pain, hypotension, bradycardia, and the potential for respiratory depression. Some studies even indicate that propofol may lead to a higher mortality rate when compared to other sedative agents [4]. Moreover, no specific antagonists are available to counteract propofol’s sedative effects [5].

Remimazolam, a novel ultra-short-acting benzodiazepine, exerts sedative and hypnotic effects by targeting γ-aminobutyric acid type A (GABA_A_) receptors and undergoes rapid conversion into inactive metabolites through tissue esterase enzymes [6]. Remimazolam offers advantages over midazolam, including a quicker onset of action, swifter recovery, and better controllability [7,8]. In comparison to propofol, remimazolam exhibits milder effects on circulation and respiration, with rare occurrences of side effects like injection pain [9,10,11,12]. Notably, the sedative effects of remimazolam can be selectively reversed by flumazenil [13,14], a capability not offered by propofol [5].

Flumazenil, a specific benzodiazepine receptor antagonist, can reverse the cognitive, psychomotor, hypnotic, and electroencephalographic effects of remimazolam by competitively antagonizing GABA_A_ receptors [15]. Nevertheless, it appears ineffective in reversing the amnestic effect [13]. Because it is rapidly metabolized by the liver, the antagonism of flumazenil after a single intravenous injection lasts only 30 to 60 min, after which supplementary doses may be required to maintain an ideal level of consciousness [16].

Several studies and meta-analyses have suggested that postoperative recovery with remimazolam may not be faster than that with propofol. [17,18,19,20,21]. However, the recovery from sedation and anesthesia with remimazolam, when used alongside flumazenil, may be faster than with propofol, and a systematic review on this topic is still pending. This study aimed to conduct a systematic review and meta-analysis to examine the hypotheses mentioned above, all the while addressing concerns regarding re-sedation.

## 2. Materials and Methods

### 2.1. Overview

This systematic review and meta-analysis were conducted following the methodologies recommended by the Cochrane Collaboration and documented following the PRISMA (Preferred Reporting Items for Systematic Reviews and Meta-Analyses) 2020 statement [22] (S2). The protocol has been registered on the PROSPERO website (ID: CRD42023462788).

### 2.2. Search Strategy

Electronic databases, including PubMed, Embase, Web of Science, and the Cochrane Library, were systematically searched from their inception up to 22 October 2023. We employed Boolean operators to combine Medical Subject Headings (MeSH) such as ‘remimazolam’, ‘flumazenil’, and ‘propofol’ along with their respective synonyms, in our search across designated databases. Filters were applied to refine the selection of relevant study types. The comprehensive search strategies for each database are available in the Supplementary Materials (S1). Furthermore, we manually retrieved citations from the included articles and relevant research to identify potentially eligible studies.

### 2.3. Selection Process and Criteria

Two reviewers (QW and JW) independently conducted the search, removed duplicate articles using automated procedures and manual intervention, screened the remaining articles by reviewing titles and abstracts, and assessed the full text of the remaining articles to determine their final inclusion. Any discrepancies that arose during the screening process were resolved through discussion to reach a consensus; if needed, a decision was deferred to another author (MJ).

Studies meeting the following criteria were included: (1) Participants: adult patients receiving procedural sedation or general anesthesia and returning to the ward. (2) Interventions: the administration of remimazolam as the intravenous anesthetic, with the routine use of flumazenil to reverse the anesthesia upon completion. (3) Controls: the use of propofol as the intravenous anesthetic. (4) Outcomes: Assessment of postoperative recovery conditions, including emergence time, extubation time, length of stay in the post-anesthesia care unit (PACU), and the occurrence of postoperative adverse events. (5) Study design: Inclusion criteria were limited to clinical randomized controlled trials (RCTs). (6) Studies must be published or accepted in a peer-reviewed journal with full-text availability. As we anticipated a limited number of eligible studies, we refrained from imposing language restrictions. Additionally, we included studies where flumazenil was routinely administered in the control group because previous research has indicated a minimal impact of flumazenil on propofol [5,23].

### 2.4. Data Extraction

Data extraction was independently conducted by two reviewers (QW and FX) from the included articles and recorded in a dedicated spreadsheet. If any essential data were missing in an article meeting inclusion criteria, the reviewers contacted the authors via email to request the necessary information. Discrepancies during data extraction were resolved through consensus discussions. The following information was collected from each selected article: first author, publication year, geographical location, study design, grouping and intervention methods, sample size, participant demographics, procedure or surgery details, anesthesia protocol, primary and secondary outcomes, measurement methods, and corresponding data.

The primary outcome in this study, emergence time, exhibited variations in its definition across the included trials. We attempted to choose the definition adopted by most of the selected studies, i.e., eye opening time, as the emergence time. In cases where the study did not measure eye opening time, we extracted the most closely related outcome (such as the time to obey verbal commands) and incorporated it into the meta-analysis. Some of the selected studies provided data on the length of PACU stay, while others solely presented the time taken to meet PACU discharge criteria, specifically achieving an Aldrete score of ≥9 [24,25]. We incorporated both sets of data into our meta-analysis.

### 2.5. Risk of Bias Assessment

Two independent reviewers (QW and FX) utilized the updated Cochrane risk of bias tool for randomized trials (RoB 2), last revised on 22 August 2019, to evaluate the quality of the included RCTs [26]. The quality of each RCT was assessed by answering signaling questions within five domains: the randomization process, deviations from intended interventions, missing outcome data, measurement of the outcome, and selection of the reported result. Using the tool, risk within each domain was automatically determined based on the responses to signaling questions, resulting in classifications of low risk, some concerns, or high risk. The overall bias rating aligned with the domain with the highest risk. In cases where the automatically determined assessment appeared unreasonable, the evaluators had the discretion to make an independent judgment and must provide accompanying reasons for subsequent discussion and consensus. In cases of discrepancies, consensus was reached through discussion. Assessment results were visually presented using Review Manager software (Version 5.4, The Cochrane Collaboration, 2020).

### 2.6. Data Synthesis and Analysis

Data synthesis and analysis were conducted using Stata 17 software (StataCorp. 2021. Stata Statistical Software: Release 17. College Station, TX, USA: StataCorp LLC). For continuous variables, medians (interquartile range, IQR) were transformed into means ± standard deviations (SDs) following the approach described by Wan et al. [27]. Effect sizes were expressed as mean differences (MDs) with their respective 95% confidence intervals (CIs) for continuous outcomes and as risk ratios (RRs), with corresponding 95% CIs for dichotomous variables. All hypothesis tests were two-sided. When the 95% CIs of MDs included 0 or the 95% CIs of RRs included 1, the difference was considered statistically nonsignificant. The results of the meta-analyses were summarized and visually presented in forest plots.

### 2.7. Heterogeneity Treatment and Meta-Regression

Heterogeneity among the included studies was assessed using both the Cochrane Q test and the *I*² test [28]. The *I*² test quantified heterogeneity using the formula *I*² = (Q − df)/Q × 100%, where Q represented Cochran’s heterogeneity statistic and df stood for degrees of freedom [29]. If *I*² was less than 50%, indicating mild heterogeneity, the data were combined using a fixed-effects model. Conversely, if significant heterogeneity was observed, a random-effects model was applied. In cases where the number of included studies was limited, and there was substantial heterogeneity, we employed the Hartung–Knapp–Sidik–Jonkman method for random-effects meta-analysis to mitigate the risk of obtaining erroneous results [30].

To address significant heterogeneity in the primary outcome, we identified relevant variables in each study. We conducted univariate meta-regression to assess their correlation with the meta-analysis results, measured using R^2^. Variables with R^2^ > 0 were then included in a multivariable meta-regression to explore the primary sources of heterogeneity, which guided the formation of subgroups.

### 2.8. Sensitivity Analysis

The “leave-one-out” meta-analyses were employed to validate the reliability and stability of the results. The “leave-one-out” meta-analysis assessed the robustness of the results by systematically excluding each study one at a time and observing its impact on statistical significance. The results of the “leave-one-out” meta-analyses were summarized and visually presented in forest plots.

### 2.9. Publication Bias

Since the number of included studies was fewer than 10, we only assessed publication bias for the primary outcome. We employed a funnel plot for qualitative detection and the Egger test for quantitative detection. If necessary, we used the trim-and-fill procedure to evaluate the results adjusted for publication bias.

### 2.10. Certainty of Evidence

The quality of evidence for each outcome was independently assessed by two reviewers (QW and JW) following the approach recommended by the Grading of Recommendations, Assessment, Development, and Evaluation (GRADE) Working Group [31]. Any disagreements were discussed to achieve consensus. Elaboration on the assessment of evidence certainty can be found in the Supplementary Materials (S3).

## 3. Results

### 3.1. Search and Selection

**Figure 1:**
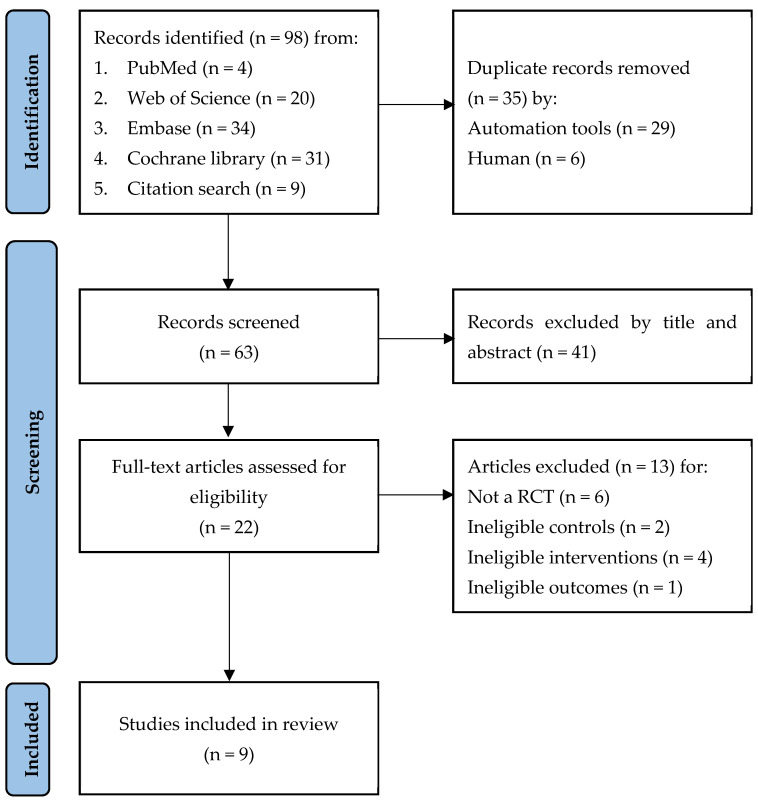
The PRISMA flow diagram of the selection process.

A total of 98 relevant records were initially retrieved from the designated databases, and 9 of these were identified through manual citation searches. Following the removal of 35 duplicate records through automated and manual methods, an additional 41 records were excluded after reviewing their titles and abstracts, as they did not meet the inclusion criteria. Upon reviewing the full-text articles of the remaining records, we excluded 13 studies for various reasons: they were not randomized controlled trials [32,33,34,35,36,37], did not set up a propofol group [38,39], did not have routine flumazenil usage in the remimazolam group [19,40,41,42], or did not have outcomes we wanted [43]. Ultimately, nine studies meeting the criteria were included [44,45,46,47,48,49,50,51,52]. The selection process is illustrated in Figure 1.

### 3.2. Study Characteristics

After meticulous screening, a total of nine RCTs involving 745 patients from three countries were included in this review. We categorized the baseline characteristics of the included studies into individual-level and study-level characteristics, as presented in Table 1 and Table 2, respectively.

At the individual level, the mean age of participants in each study ranged from 41.7 to 75.04. Each study included both males and females, with patients’ American Society of Anesthesiologists (ASA) Classification ranging from 1 to 4. At the study level, each of the nine trials involved different procedures, but all patients received general anesthesia with either a laryngeal mask airway (LMA) [46,47,48] or tracheal intubation [44,45,49,50,51,52]. Eight studies administered flumazenil routinely after discontinuation of remimazolam, and one study had two remimazolam groups [46], one with flumazenil and one without. Additionally, two studies used flumazenil after discontinuation of propofol [49,52]. In the included studies, flumazenil was administered immediately after remimazolam discontinuation [45,47,49,50,51,52], 10 min after remimazolam discontinuation [46], when the Train of Four (TOF) > 90% [48], or upon the restoration of spontaneous breathing [44]. Following the discontinuation of anesthesia, seven studies employed neuromuscular blockade reversal agents [44,45,46,47,48,49,51], with only one study utilizing opiate receptor antagonists [46].

### 3.3. Risk of Bias

**Figure 2:**
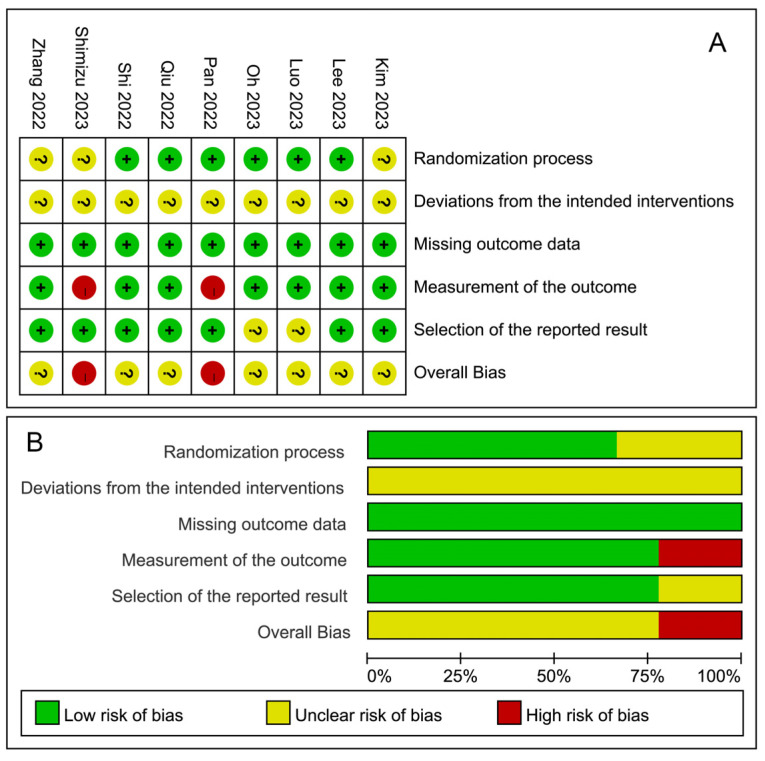
Assessment of risk of bias; (**A**) risk of bias for each study; (**B**) risk of bias summary [44,45,46,47,48,49,50,51,52].

Assessments of the risk of bias are presented in Figure 2. Regarding the randomization process, three studies were assessed as having some concerns due to a lack of detailed information on allocation concealment [44,51,52], while the remaining studies were assessed as low risk. Regarding deviations from the intended interventions, all the studies were not blinded to the attending anesthesiologists, which may cause bias. Therefore, all studies were evaluated as having some concerns in this domain. In terms of outcome measurement, two studies were deemed high risk due to the potential lack of blinding among outcome assessors [48,51]. After a thorough comparison with the trial registration, two studies were assessed as having some concerns regarding the selection of the reported result [46,47]. The overall bias rating aligned with the domain with the highest risk.

### 3.4. Meta-Analysis Results

#### 3.4.1. Emergence Time

**Figure 3:**
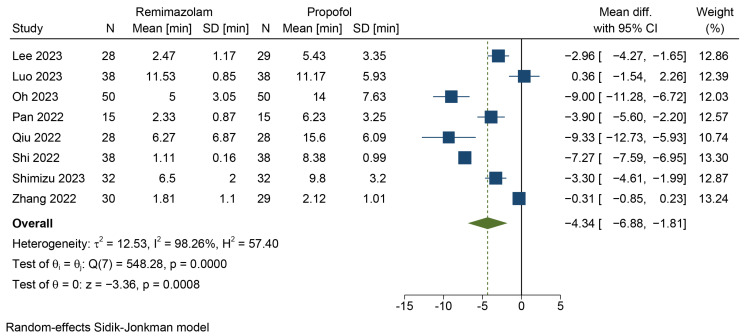
Forest plot for emergence time [45,46,47,48,49,50,51,52].

Data regarding emergence time were extracted from eight studies [45,46,47,48,49,50,51,52], encompassing 518 patients from three different countries. A random-effects model and the Hartung–Knapp–Sidik–Jonkman method were employed for data synthesis, showing faster emergence in the remimazolam–flumazenil group compared to propofol (MD = −4.34 min, 95% CI = [−6.88, −1.81], *p* = 0.0008, *I*^2^ = 98.26%) (Figure 3). The quality of evidence was low.

**Figure 4:**
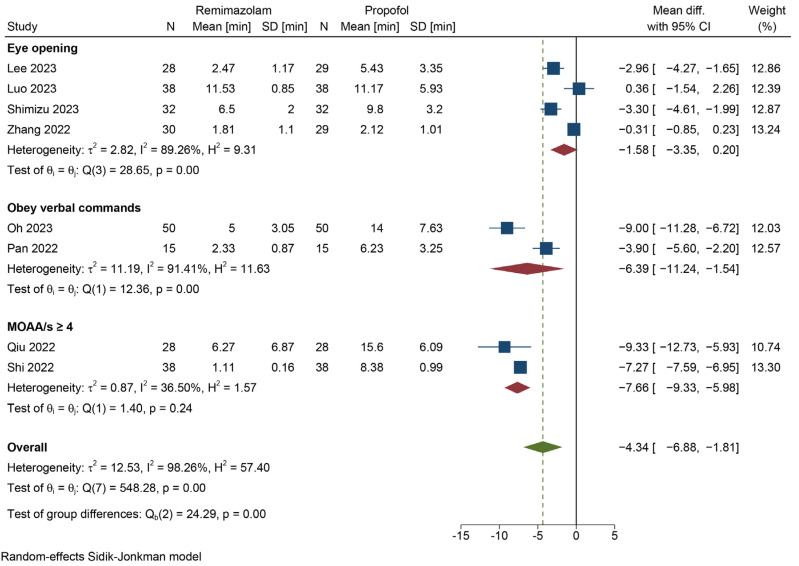
Subgroup analysis for emergence time [45,46,47,48,49,50,51,52].

To address the high heterogeneity, we extracted potential contributing independent variables from each study, including individual-level and study-level factors. Univariate meta-regression was employed to assess their associations with the meta-analysis outcomes, as detailed in Table 3. Variables with R^2^ > 0 were subsequently included in a multivariable meta-regression analysis, as presented in Table 4. The meta-regression results indicated that the definition of emergence was a significant moderator, independently accounting for 62.29% of the observed heterogeneity. Given these findings, we conducted a subgroup analysis based on the definition of emergence, with the results displayed in Figure 4. When eye opening was utilized as the definition of emergence, there was no statistically significant difference in emergence time between the remimazolam–flumazenil group and the propofol group (MD = −1.58 min, 95% CI = [−3.35, 0.2]). The results of the remaining subgroups were consistent with the overall results, indicating that the remimazolam–flumazenil group provided a shorter emergence time. The heterogeneity of each subgroup was lower than the total, and there was significant heterogeneity between subgroups (*p* < 0.01), further verifying that the definition of emergence was the primary source of heterogeneity.

#### 3.4.2. Extubation Time

**Figure 5:**
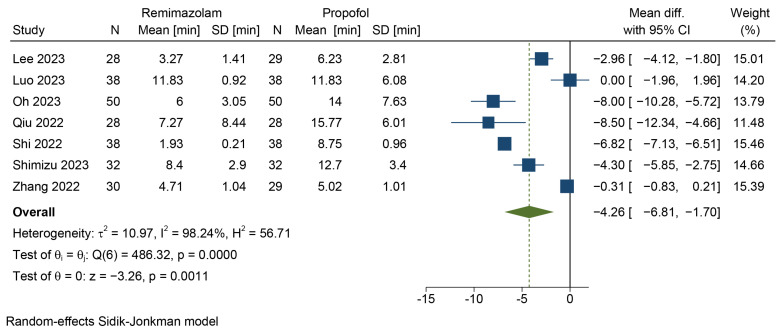
Forest plot for extubation time [45,46,47,49,50,51,52].

Seven studies involving 488 patients measured the time to extubation or LMA removal [45,46,47,49,50,51,52]. After pooling the data using the Hartung–Knapp–Sidik–Jonkman method and the random-effects model, the results are presented in Figure 5. The meta-analysis results indicated that the combination of remimazolam and flumazenil resulted in a shorter extubation time compared to propofol (MD = −4.26 min, 95% CI = [−6.81, −1.7], *p* = 0.0011, *I*^2^ = 98.24%). The quality of evidence was low.

#### 3.4.3. Length of PACU Stay

**Figure 6:**
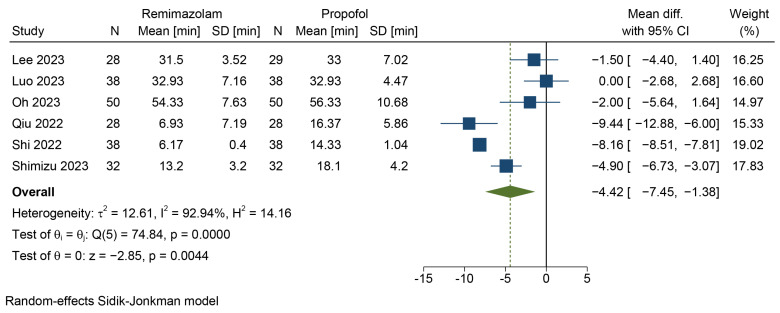
Forest plot for the length of PACU stay [45,46,47,49,50,51].

Four studies reported the length of the PACU stay for 297 patients [45,47,50,51], while two studies observed the time it took for 132 patients to meet the PACU discharge criteria [46,49]. The random-effects model and the Hartung–Knapp–Sidik–Jonkman method were employed to aggregate the data, and the results are provided in Figure 6. Remimazolam in combination with flumazenil resulted in a shorter PACU stay when compared to propofol (MD = −4.42 min, 95% CI = [−7.45, −1.38], *p* = 0.0044, *I*^2^ = 92.94%). The quality of evidence was low.

#### 3.4.4. Postoperative Complications

We collected data on postoperative complications from all the selected studies and performed separate meta-analyses for each complication. The comprehensive analysis results for each complication are presented in Table 5.

All complications were analyzed using fixed-effects models in the meta-analyses. There were no significant differences observed in postoperative pain, postoperative nausea and vomiting (PONV), emergence agitation, delirium, or dizziness between the two anesthesia regimens. The remimazolam group exhibited a lower incidence of respiratory depression compared to the propofol group (0% vs. 5.16%, RR = 0.2, 95% CI = [0.04, 0.89], *p* = 0.03, *I*^2^ = 0). However, the incidence of re-sedation following the combined use of remimazolam and flumazenil was significantly higher than with propofol (9.03% vs. 1.38%, RR = 4.15, 95% CI = [1.31, 13.13], *p* = 0.01, *I*^2^ = 35.07%). The quality of evidence for each outcome is also provided in Table 5.

### 3.5. Sensitivity Analysis

**Figure 7:**
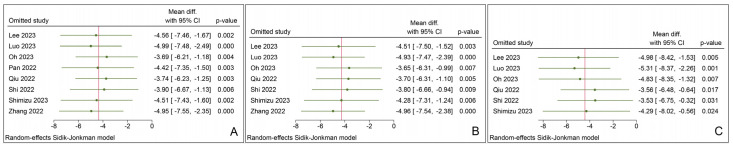
“Leave-one-out” sensitivity analysis for emergence time (**A**), extubation time (**B**), and length of PACU stay (**C**) [45,46,47,48,49,50,51,52].

We conducted “leave-one-out” sensitivity analyses for the outcomes of emergence time, extubation time, and the length of PACU stay to assess the robustness of these results in the presence of high heterogeneity. The removal of any single study did not alter the statistical significance of the three overall outcomes, as demonstrated in Figure 7. This suggests that these outcomes are robust.

### 3.6. Publication Bias

**Figure 8:**
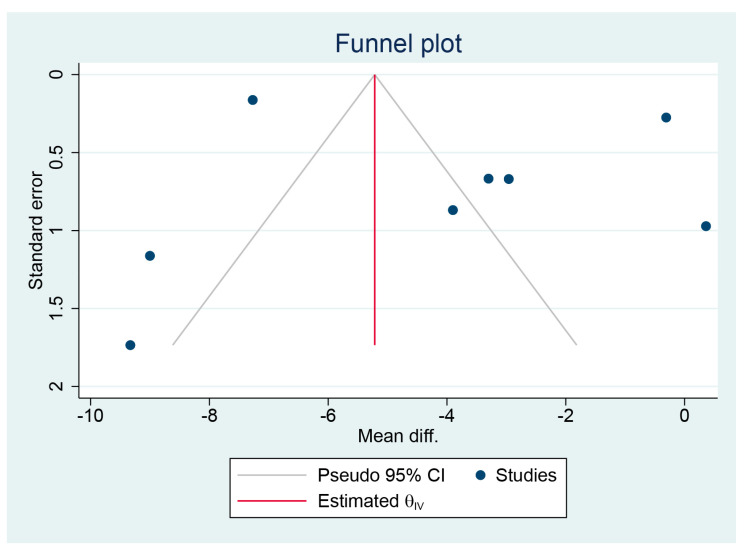
Funnel plot for emergence time.

Considering the limited number of studies included in this systematic review, we solely evaluated publication bias for the primary outcome, which was the emergence time in the meta-analysis. A funnel plot was employed for qualitative analysis, as depicted in Figure 8. Additionally, the Egger test was conducted for quantitative analysis, indicating no evidence of publication bias related to emergence time (*p* = 0.245), as shown in the Supplementary Materials (S4).

## 4. Discussion

We conducted a systematic review and meta-analysis comparing remimazolam–flumazenil and propofol for general anesthesia recovery for the first time. Our findings showed that remimazolam–flumazenil resulted in faster emergence and extubation, a shorter PACU stay, and a lower risk of respiratory depression compared to propofol. However, it came with a higher re-sedation risk.

Our primary outcome exhibited significant heterogeneity. To explore the sources of this heterogeneity, we conducted a meta-regression analysis, considering various independent variables. The results identified the definition of emergence as the primary contributor to the heterogeneity. Subsequent subgroup analysis confirmed this finding, while other factors such as patients’ mean age, surgical duration, intubation or LMA usage, the administration of neuromuscular blockade reversal agents, and opioid receptor antagonists did not significantly impact the heterogeneity. Interpreting the results of meta-regression with caution is essential given the limited number of studies included in the analysis.

Recent studies have suggested that remimazolam could lead to a longer recovery from anesthesia compared to propofol [42,53,54,55,56]. Additionally, the meta-analysis of Zhang et al. indicated a longer awakening time following general anesthesia with remimazolam compared to propofol [20]. One significant advantage of remimazolam is its reversibility with flumazenil. Nevertheless, none of the aforementioned studies assessed the effect of remimazolam in combination with flumazenil. In our meta-analysis, which encompassed RCTs comparing remimazolam–flumazenil and propofol for general anesthesia, we found that the use of flumazenil following remimazolam led to a shorter recovery compared to propofol.

While flumazenil effectively reverses the effects of remimazolam, several studies have reported the occurrence of re-sedation following its administration [57,58,59]. Our findings indicated that the use of remimazolam in combination with flumazenil did carry a higher risk of re-sedation (9.03%) compared to propofol (1.38%). Some researchers have proposed that factors such as a high bolus dose of flumazenil and reduced total remimazolam clearance may contribute to this phenomenon [57]. Furthermore, considering flumazenil’s rapid hepatic metabolism [15,16], it is plausible that flumazenil is eliminated more swiftly than remimazolam, which could potentially contribute to the occurrence of re-sedation. However, the exact mechanism of re-sedation following flumazenil administration remains unclear.

In recent years, Enhanced Recovery After Surgery (ERAS) programs have gained global popularity due to their objectives of reducing complications, shortening hospital stays, and cutting medical costs [60]. ERAS implementation places greater demands on anesthesia, emphasizing the minimization of perioperative complications, early recovery and extubation, and swift return to the ward [61]. Prior research has indicated that remimazolam offers advantages over propofol, such as more stable hemodynamics [9], reduced respiratory depression [10], and fewer complications [12]. Nevertheless, there has been ongoing debate about its impact on postoperative recovery and discharge times.

Our findings indicated that, in comparison to propofol, general anesthesia with remimazolam–flumazenil led to a reduction in emergence time by 4.34 min, extubation time by 4.26 min, and PACU discharge time by 4.42 min. These results can be particularly beneficial for the successful implementation of ERAS, especially in day surgery settings. However, it is worth noting that remimazolam–flumazenil carried a higher risk of re-sedation, based on data collected during the PACU period. Whether re-sedation remains a concern after patients return to the ward is a topic that merits further exploration. Therefore, when considering the use of remimazolam combined with flumazenil in clinical practice, a thorough evaluation of its advantages and disadvantages is essential.

### 4.1. Limitations

Although we employed scientific methods for our systematic review and meta-analysis, there were still certain limitations present. First, it is important to note that the overall quality of the included studies in our analysis was relatively low. This is primarily due to the substantial differences in the appearance and dosage of the two drugs, making it difficult to blind the interventionists and outcome assessors, which is a key factor contributing to the reduced quality of the evidence. Secondly, our initial intent was to include studies on both procedural sedation and general anesthesia. However, we did not identify any studies related to procedural sedation in the screening results. Furthermore, it is important to note that the studies included in our analysis were conducted exclusively in three East Asian countries. This limited geographical diversity may impact the generalizability of our findings to a broader population. Methodologically, certain results in our study exhibited significant heterogeneity. Despite our efforts to identify and address the primary sources of heterogeneity through meta-regression, subgroup heterogeneity remained relatively high. Fortunately, our sensitivity analysis confirmed the stability of our results.

### 4.2. Strengths and Future Directions

This study employed rigorous systematic review and meta-analysis methodologies to address important clinical issues that have received limited attention. We conducted a meticulous and impartial review of existing research and data in compliance with the PRISMA statement. Furthermore, we assessed the level of evidence certainty for each outcome. Our study holds the potential to offer multifaceted insights for the clinical utilization of remimazolam.

For future research directions, aside from continuing to conduct high-quality similar research, it is imperative to direct attention towards the utilization of remimazolam–flumazenil in procedural sedation. Furthermore, there is a need for additional research to delve into the underlying mechanisms of re-sedation, the potential for re-sedation post-PACU discharge, and strategies for re-sedation prevention.

## 5. Conclusions

In summary, when compared to propofol, the combination of remimazolam and flumazenil accelerated emergence, extubation, and PACU discharge following general anesthesia. Additionally, remimazolam–flumazenil reduced the incidence of respiratory depression during the recovery phase but posed a higher risk of re-sedation. However, due to limitations in the quality of the evidence, it is advisable to interpret the results of meta-analyses with caution. In the future, it is imperative to conduct numerous well-designed and homogeneous studies to validate the findings of this article. Moreover, to better understand the risk of re-sedation associated with remimazolam, future studies with larger sample sizes should be designed to quantify this risk more precisely. Additionally, studies that investigate the occurrence and implications of re-sedation upon returning to the ward should be conducted. Further exploration of the risk factors, underlying mechanisms, and prevention strategies of re-sedation is also warranted.

## Figures and Tables

**Table 1:** Individual-level characteristics of included studies.

Study Year	Group-ing	Age (Years)	Gender (F/M)	ASA Class	Duration of Surgery (Min)	Duration of Anesthesia (Min)
Kim [44] 2023	RF	41.7 ± 12.2	33/61	1-2	42.8 ± 24.5	71.8 ± 26.1
	P	43.3 ± 13.2	36/59	1-2	46.4 ± 22.9	75 ± 22.4
Lee [45] 2023	RF	45 ± 13.4	21/7	1-2	85 (70, 98)	NR
	P	51 ± 12.1	19/10	1-2	85 (75, 105)	NR
Luo [46] 2023	RF	44.7 ± 16.8	16/22	1-2	38.6 ± 29.1	NR
	R	43.5 ± 15.6	24/14	1-2	42.2 ± 27.7	NR
	P	44.3 ± 18.1	19/19	1-2	38.7 ± 25.1	NR
Oh [47] 2023	RF	60 (54, 65)	8/42	2-3	NR	124 (112, 142)
	P	60 (52, 64)	8/42	2-3	NR	123 (116, 140)
Pan [48] 2022	RF	61.13 ± 8.62	3/12	2-4	36.67 ± 19.85	41.47 ± 19.31
	P	60.13 ± 7.24	0/15	2-3	44.40 ± 22.72	49.60 ± 22.86
Qiu [49] 2022	RF	62.8 ± 7.1	7/21	1-3	53 (27.5, 81)	92.5 (66.3, 120.8)
	PF	64.7 ± 8.9	9/19	1-3	55 (35.5, 77.3)	87 (71.5, 114.8)
Shi [50] 2022	RF	52.74 ± 4.93	22/16	2-3	27 ± 2.72	NR
	P	51.61 ± 5.48	20/18	2-3	26.88 ± 2.88	NR
Shimizu [51] 2023	RF	43.5 ± 10.4	10/22	1-2	107 ± 38	157 ± 42
	P	44.4 ± 10.1	10/22	1-2	121 ± 57	178 ± 60
Zhang [52] 2022	RF	74.31 ± 10.6	19/11	2-3	NR	130.16 ± 43.01
	PF	75.04 ± 9.98	17/12	2-3	NR	131.64 ± 45.63

RF: remimazolam–flumazenil; P: propofol; R: remimazolam; PF: propofol-flumazenil; F: female; M: male; ASA: American Society of Anesthesiologists; NR: not reported. Continuous data are presented as mean ± standard deviation or median (interquartile range).

**Table 2:** Study-level characteristics of included studies.

Study Year	Country	Grouping	Sample Size	RB/RT	Anesthetic Dose		Flumazenil Dose	FDT	Surgery	Airway	NMBRA	ORA
					Induction	Maintenance						
Kim [44] 2023	Korea	RF	94	NR	12 mg/kg/h	1–2 mg/kg/h	0.2 mg	➃	OMS	Intubation	Yes	No
		P	95		TCI 3–5 μg/mL	TCI 3–5 μg/mL	―					
Lee [45] 2023	Korea	RF	28	RB	6 mg/kg/h	1–2 mg/kg/h	0.2–1 mg	➀	TT	Intubation	Yes	No
		P	29		TCI 3 ng/mL	TCI ≥ 2 ng/mL	―					
Luo [46] 2023	China	RF	38	RT	0.3 mg/kg	1–3 mg/kg/h	0.2–1 mg	➁	DS	LMA	Yes	Yes
		R	38		0.3 mg/kg	1–3 mg/kg/h	―					
		P	38		2–2.5 mg/kg	6–12 mg/kg/h	―					
Oh [47] 2023	Korea	RF	50	RB	6 mg/kg/h	1–2 mg/kg/h	0.2–1 mg	➀	CA	LMA	Yes	No
		P	50		TCI 5 μg/mL	TCI 3–5 μg/mL	―					
Pan [48] 2022	China	RF	15	RB	0.4 mg/kg	1 mg/kg/h	0.5 mg	➂	RBS	LMA	Yes	No
		P	15		1.5 mg/kg	4–8 mg/kg/h	―					
Qiu [49] 2022	China	RF	28	RT	0.3 mg/kg	1–3 mg/kg/h	0.5 mg	➀	ESD	Intubation	Yes	No
		PF	28		2 mg/kg	5 mg/kg/h	0.5 mg					
Shi [50] 2022	China	RF	38	RT	0.2 mg/kg	1–2 mg/kg/h	0.5 mg	➀	EVL	Intubation	No	No
		P	38		2 mg/kg	4–10 mg/kg/h	―					
Shimizu [51] 2023	Japan	RF	32	NR	12 mg/kg/h	1–2 mg/kg/h	0.2–0.5 mg	➀	ESS	Intubation	Yes	No
		P	32		TCI 3–4 μg/mL	TCI 2–5 μg/mL	―					
Zhang [52] 2022	China	RF	30	NR	0.2–0.4 mg/kg	0.3–0.5 mg/kg/h	0.3 mg	➀	HR	Intubation	No	No
		PF	29		1.5–2 mg/kg	4–8 mg/kg/h	0.3 mg					

RF: remimazolam–flumazenil; P: propofol; R: remimazolam; PF: propofol-flumazenil; RB: remimazolam besylate; RT: remimazolam tosilate; NR: not reported; TCI: target-controlled infusion; FDT: flumazenil dosing time; OMS: oral and maxillofacial surgery; TT: thyroidectomy; DS: day surgery; CA: catheter ablation; RBS: rigid bronchoscopy surgery; ESD: endoscopic submucosal dissection; EVL: endoscopic variceal ligation; ESS: endoscopic sinus surgery; HR: hip replacement; LMA: laryngeal mask airway; NMBRA: neuromuscular blockade reversal agent; ORA: opioid receptor antagonist; ➀: immediately after anesthesia discontinuation; ➁: 10 min after anesthesia discontinuation; ➂: the Train of Four > 90%; ➃: restoration of spontaneous breathing.

**Table 3:** Univariable meta-regressions.

Variable	β	z	*p* Value	95% CI	R^2^ (%)
Individual-level variables					
Mean age	−0.04	−0.31	0.76	−0.31, 0.22	0
ASA class 1–2 (%)	0.01	0.15	0.88	−0.12, 0.14	0
Surgery duration	0.01	0.28	0.78	−0.06, 0.08	0
Female (%)	0.08	1.34	0.18	−0.04, 0.19	10.47
Mean BMI	−0.52	−0.43	0.66	−2.87, 1.82	0
Study-level variables					
Country					0
China/others	−0.67	−0.15	0.88	−9.48, 8.13	
Korea/others	−2.6	−0.52	0.6	−12.47, 7.27	
Control (P/PF)	0.07	0.02	0.98	−6.3, 6.44	0
RB/RT	0.08	0.02	0.98	−6.63, 6.78	0
Flumazenil dose	−10.83	−1.19	0.23	−28.65, 6.99	5.68
FDT (➀/others)	−3.40	−1.17	0.24	−9.11, 2.31	4.94
Airway (LMA/intubation)	0.35	0.12	0.9	−5.28, 5.98	0
NMBRA (yes/no)	0.76	0.24	0.8	−5.39, 6.92	0
ORA (yes/no)	−5.35	−1.47	0.14	−12.5, 1.8	14.43
Primary outcome (yes/no)	−0.83	−0.3	0.76	−6.24, 4.58	0
Sample size	−0.05	−0.77	0.44	−0.19, 0.08	0
Emergence definition					62.29
Eye opening/others	6.48	3.15	0.002	2.45, 10.52	
Obey verbal commands /others	1.73	0.72	0.47	−2.99, 6.46	
Risk of bias (some concerns/high risk)	−1.02	−0.32	0.75	−7.23, 5.19	0

β: coefficient; CI: confidence interval; ASA: American Society of Anesthesiologists; BMI: body mass index; P: propofol; PF: propofol-flumazenil; RB: remimazolam besylate; RT: remimazolam tosilate; FDT: flumazenil dosing time; ➀: immediately after anesthesia discontinuation; LMA: laryngeal mask airway; NMBRA: neuromuscular blockade reversal agent; ORA: opioid receptor antagonist.

**Table 4:** Multivariable meta-regression.

Variable	β	z	*p* Value	95% CI
Emergence definition				
Eye opening/others	12.28	3.13	0.002	4.6, 19.96
Obey verbal commands/others	7.22	1.62	0.1	−1.52, 15.96
ORA (yes/no)	−5.96	−1.08	0.28	−16.73, 4.81
Female (%)	0.02	0.6	0.55	−0.05, 0.1
Flumazenil dose	25.78	1.82	0.07	−2.03, 53.6
FDT (➀/others)	2.49	0.52	0.6	−6.89, 11.88

β: coefficient; CI: confidence interval; ORA: opioid receptor antagonist; FDT: flumazenil dosing time; ➀: immediately after anesthesia discontinuation.

**Table 5:** Meta-analysis for postoperative complications (remimazolam–flumazenil vs. propofol).

Postoperative Complication	No of Studies	No of Patients	Effect Model	Method	*I*^2^ (%)	Results of Meta-Analyses		Certainty
						MD (95% CI)	*p* Value	
Pain	2 ^!^	135	Fixed	I-V	0	−0.01 (−0.08, 0.06)	0.78	High
						RR (95% CI)	*p* value	
PONV	7	587	Fixed	M-H	0	0.87 (0.49, 1.56)	0.64	High
Respiratory depression	4	311	Fixed	M-H	0	0.2 (0.04, 0.89)	0.03 *	High
Emergence agitation	4	291	Fixed	M-H	0	0.45 (0.1, 1.94)	0.28	Moderate
Re-sedation	4	289	Fixed	M-H	35.07	4.15 (1.31, 13.13)	0.01 *	Moderate
Delirium	3	172	Fixed	M-H	0	0.67 (0.11, 3.87)	0.65	Low
Dizziness	2	135	Fixed	M-H	0	0.42 (0.11, 1.57)	0.2	Moderate

^!^: A study was excluded because it reported the median and interquartile range for postoperative pain scores [44], which, after data transformation, yielded a standard deviation of 0; *: *p* < 0.05; MD: mean difference; RR: risk ratio; CI: confidence interval; I-V: Inverse-Variance; M-H: Mantel–Haenszel; PONV: postoperative nausea and vomiting.

## Data Availability

The first author of this study can provide the data upon request.

## Supplementary Materials

The following supporting information can be downloaded at: https://www.mdpi.com/article/10.3390/jcm12237316/s1, S1: the complete search strategy for each database; S2: PRISMA 2020 Checklist; S3: detailed results of the GRADE assessment; S4: Egger test of the primary outcome.
